# Synergism of Holmium Orthovanadate/Phosphorus-Doped Carbon Nitride Nanocomposite: Nonenzymatic Electrochemical Detection of Hydrogen Peroxide

**DOI:** 10.1021/acs.inorgchem.3c03804

**Published:** 2024-01-29

**Authors:** Thangavelu Kokulnathan, Tzyy-Jiann Wang, Faheem Ahmed, Thamraa Alshahrani, Nishat Arshi

**Affiliations:** † Department of Electro-Optical Engineering, National Taipei University of Technology, Taipei 106, Taiwan; ‡ Department of Applied Sciences & Humanities, Faculty of Engineering & Technology, Jamia Millia Islamia, New Delhi 110025, India; § Department of Physics, College of Science, Princess Nourah bint Abdulrahman University, P.O. Box 84428, Riyadh 11671, Saudi Arabia; ∥ Department of Basic Sciences, Preparatory Year Deanship, King Faisal University, P.O. Box-400, Al-Ahsa 31982, Saudi Arabia

## Abstract

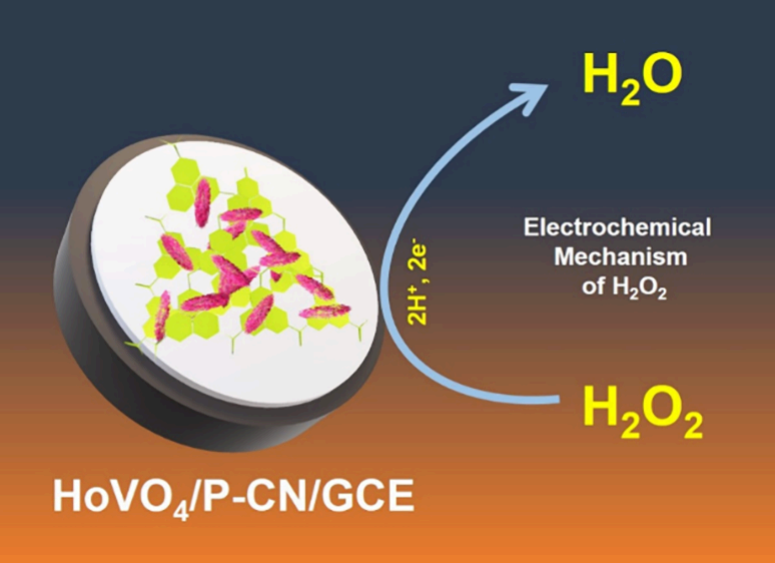

Developing efficient and robust electrode materials for electrochemical sensors is critical for real-time analysis. In this paper, a hierarchical holmium vanadate/phosphorus-doped graphitic carbon nitride (HoVO_4_/P-CN) nanocomposite is synthesized and used as an electrode material for electrochemical detection of hydrogen peroxide (H_2_O_2_). The HoVO_4_/P-CN nanocomposite exhibits superior electrocatalytic activity at a peak potential of −0.412 V toward H_2_O_2_ reduction in alkaline electrolytes while compared with other reported electrocatalysts. The HoVO_4_/P-CN electrochemical platform operated under the optimized conditions shows excellent analytical performance for H_2_O_2_ detection with a linear concentration range of 0.009–77.4 μM, a high sensitivity of 0.72 μA μM^–1^ cm^–2^, and a low detection limit of 3.0 nΜ. Furthermore, the HoVO_4_/P-CN-modified electrode exhibits high selectivity, remarkable stability, good repeatability, and satisfactory reproducibility in detecting H_2_O_2_. Its superior performance can be attributed to a large specific surface area, high conductivity, more active surface sites, unique structure, and synergistic action of HoVO_4_ and P-CN to benefit enhanced electrochemical activity. The proposed HoVO_4_/P-CN electrochemical platform is effectively applied to ascertain the quantity of H_2_O_2_ in food and biological samples. This work outlines a promising and effectual strategy for the sensitive electrochemical detection of H_2_O_2_ in real-world samples.

## Abstract 2

A holmium orthovanadate/phosphorus-doped carbon nitride (HoVO_4_/P-CN) nanocomposite was successfully synthesized by the sonochemical method and was utilized in the nonenzymatic electrochemical platform for detecting hydrogen peroxide (H_2_O_2_). The HoVO_4_/P-CN nanocomposite demonstrates excellent electrocatalytic performance with ultralow LOD and high sensitivity.

## 1 Introduction

Hydrogen peroxide (H_2_O_2_) is an essential member of stable reactive oxygen species and volatile inorganic compounds with strong oxidizing characteristics.^1−3^ It has been widely deployed in chemical industries, textiles, healthcare, fuel cells, food processing, and water treatments.^4−6^ Notably, low-concentration H_2_O_2_ (3–6%) is imperative in the coronavirus disease 2019 pandemic as a curative disinfectant to disinfect object surfaces, confined air, skin injury, and medical devices.^7^ In many countries, H_2_O_2_ is used in sterilizing milk and milk product packaging to preserve them.^8^ The annual H_2_O_2_ production exceeds 4 billion tons, and its global demand is expected to reach about 6 billion tons by 2024.^9^ Regular doses under the acceptable range did not have adverse effects. However, the excessive level of H_2_O_2_ may cause Alzheimer’s disease, Parkinson’s disease, diabetic nephropathy, cardiovascular diseases, cell carcinogenesis, DNA damage, neurodegeneration, malignancies, arteriosclerosis, and cancers.^10−14^

The improper discharge of H_2_O_2_ and its residues from various industrial sectors without proper treatment leads to serious environmental problems. Due to such toxicity, the U.S. Food and Drug Administration limits H_2_O_2_ residuals in packaging commodities below 0.5 ppm.^15^ The Chinese Food Sanitation Act prohibits H_2_O_2_ residues in foodstuffs and health products.^16^ Unfortunately, overuse, residue, and illegal addition of H_2_O_2_ occur frequently in many countries. For this reason, it is essential to accurately inspect the concentration level of H_2_O_2_ in food and biological samples.

Various analytical approaches are developed in detecting H_2_O_2_, which include surface-enhanced Raman spectroscopy, electrochemical detection, molecularly imprinted sensors, enzyme-linked immunoassay, photoluminescence, photoelectrochemical sensors, colorimetric detection, mass spectrometry, high-performance liquid chromatography, and colorimetric detection.^17−23^ Among these, electrochemical detection has drawn tremendous attention due to its fast response, high sensitivity, portability, high reliability, better accuracy, miniaturization, low cost, and user-friendly approach.^24−29^ Electrode materials are vital in electrochemical analysis to achieve an enhanced and high electrochemical performance. Kaplan et al. fabricated a nonenzymatic electrochemical sensor based on nickel–iron oxide@sulfur-doped reduced graphene oxide modified glassy carbon electrodes (GCEs) to detect H_2_O_2_.^30^ Xiong et al. synthesized cobalt oxide nanoparticles/porous carbon nanobox nanocomposites and used them as an electrode material for nonenzymatic electrochemical H_2_O_2_ detection.^31^ Therefore, the development of efficient electrode materials to enhance electrochemical performance for H_2_O_2_ detection is paramount.

Many research efforts have been directed toward the advancement of robust electrode constituents with markedly proficient electrocatalytic behavior tailored for use in electrochemical sensors. As electrode materials, binary metal oxides possess several advantages for electrochemical sensors over single-metal oxides as they have multiple oxidation states and high electrical conductivity.^32^ In line with the above, rare-earth orthovanadates have stimulated remarkable research interest for prospective electrode materials in electrochemical applications due to their remarkable physicochemical properties.^33−36^ Notably, holmium orthovanadate (HoVO_4_) is recognized as one capable electrode constituent possessing characteristics well suited for electrochemical sensors, such as high surface area, multiple exposed active sites, high electrical conductivity, adjustable structures, and tunable electronic structure, to show excellent electrocatalytic performance.^37,38^ These unique properties make it a high-performance electrode material and an ideal electrochemical platform for studying the structure–performance relationship during the electrocatalytic process. Sriram et al. prepared the HoVO_4_/f-boron nitride nanocomposite through an ultrasonication method to achieve efficient electrochemical sensing toward mercury ions.^39^ Although pristine HoVO_4_ has several advantages for electrochemical sensors, its low mass transfer efficiency and limited electrochemical stability are significant limitations. Therefore, to avoid these hindrances, coupling the pristine HoVO_4_ with a conductive matrix can be adopted as a valuable strategy to enhance the electrochemical properties.

Specifically, graphitic carbon nitride (g-C_3_N_4_, labeled g-CN) with a two-dimensional layered structure exhibits versatile redox properties and high durability and has been widely studied for electrochemical sensors.^40−43^ Additionally, incorporating heteroatoms into the structural framework of g-CN can considerably develop the catalytic performance of the electrochemical sensor by enhancing electron conductivity, charge distribution, desirable electronic structure, open edge sites, lattice defects, remarkable mechanical robustness, multiple chemical properties, and ample charge transportation.^44−47^ Phosphorus can be doped into the g-CN lattice (P-CN) for tuning physiochemical properties and enhancing electrocatalytic efficiency.^48^ Ahmed et al. synthesized zinc oxide/P-CN composites for electrochemical detection of nitrofurantoin, which was proven to be an efficient electrode material with good electrochemical performance.^49^ Sriram et al. reported that manganese cobalt oxide encapsulated a P-CN-modified electrode for sulfadiazine detection with high sensitivity.^50^

Inspired by the aforementioned considerations, we synthesized the HoVO_4_/P-CN nanocomposite through hydrothermal and sonochemical methods for electrochemical detection. To the best of our knowledge, this is the first report on the design and construction of HoVO_4_/P-CN nanocomposite-based electrode material applied in the nonenzymatic electrochemical H_2_O_2_ sensors. The prepared HoVO_4_/P-CN nanocomposite is methodically investigated and characterized by using several analytical techniques. The studies on cyclic voltammograms (CV) for H_2_O_2_ detection confirm that the HoVO_4_/P-CN nanocomposite-modified GCE has better electrochemical performance than the pristine HoVO_4_/GCE and P-CN/GCE due to enhanced electroactive sites, synergistic effect, fast electron/mass transfer, high conductivity, and large surface area. This excellent electrochemical performance of HoVO_4_/P-CN/GCE includes a broad detection range, low limit of detection (LOD), high steady-state stability, good selectivity, high sensitivity, and good reproducibility and repeatability. Moreover, the HoVO_4_/P-CN-based electrochemical sensor is effectually utilized for determining the H_2_O_2_ quantity in actual samples. This study provides a new perspective for constructing rare-earth orthovanadate-based electrode materials with hierarchical nanostructures for high-efficiency electrochemical sensors.

## 2 Experimental Section

Materials, instrumentation, and synthesis of HoVO_4_ nanorices and P-CN nanosheets are listed in the Supporting Information.

### 2.1 Preparation of the HoVO_4_/P-CN Nanocomposite

**Scheme 1:**
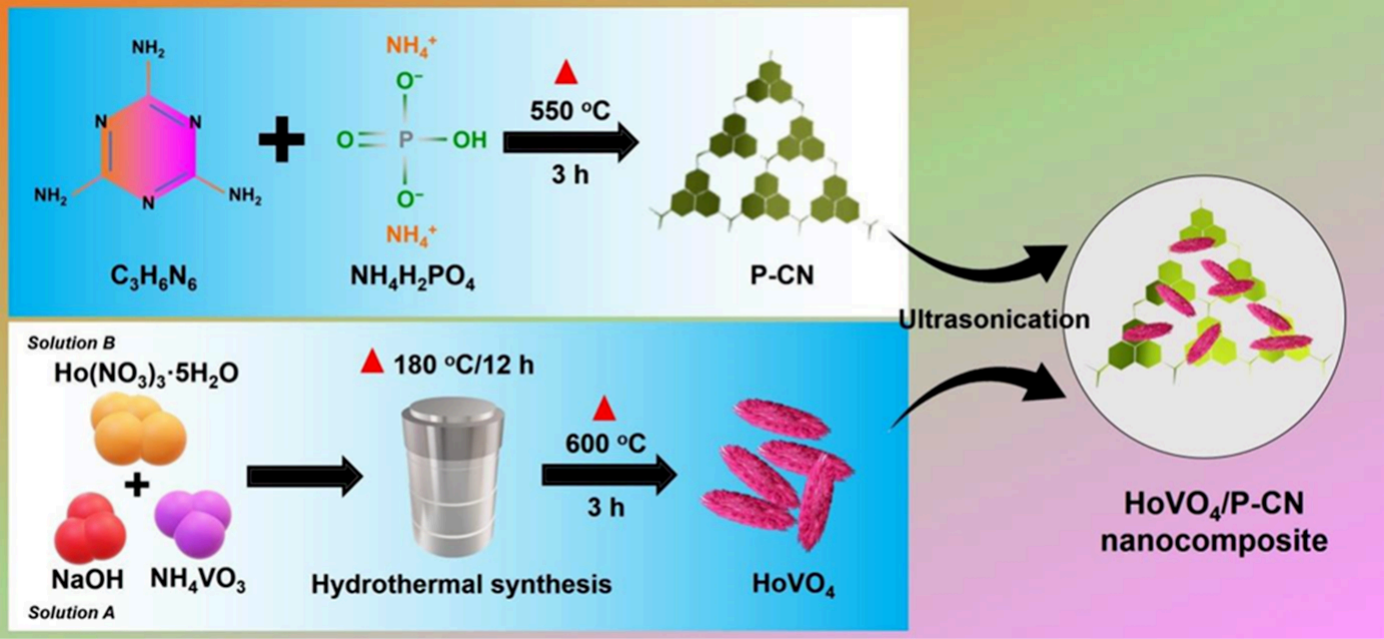
Schematic Diagram for the Preparation of the HoVO_4_/P-CN Nanocomposite

The HoVO_4_/P-CN nanocomposite was prepared by using the ultrasonic method. First, 15 mg of HoVO_4_ and 10 mg of P-CN were dispersed in 10 mL of DI water by ultrasonication for 60 min. The obtained material was centrifuged, rinsed with DI water several times, and finally dried overnight to acquire the HoVO_4_/P-CN nanocomposite. The synthesis process of the HoVO_4_/P-CN nanocomposite is schematically shown in Scheme 1.

### 2.2 Electrode Modification by HoVO_4_, P-CN, and HoVO_4_/P-CN

Before modification, the bare GCE was polished by an aqueous slurry of alumina powder with a 0.5 μm average particle size. Then, it was thoroughly rinsed in DI water and dried in air. The electrocatalyst solution was prepared by dispersing 5 mg of the HoVO_4_/P-CN nanocomposite into 1 mL of DI water under ultrasonication for 15 min to form a homogeneous suspension. Afterward, 8.0 μL of the HoVO_4_/P-CN suspension was dropped onto the surface of the pretreated GCE and dried in air. The same process was used to construct other modified electrodes, including HoVO_4_/GCE and P-CN/GCE, for comparison.

### 2.4 Safety Statement

No uncommon hazards are noted.

## 3 Result and Discussion

### 3.1 Physical Characterization

**Figure 1:**
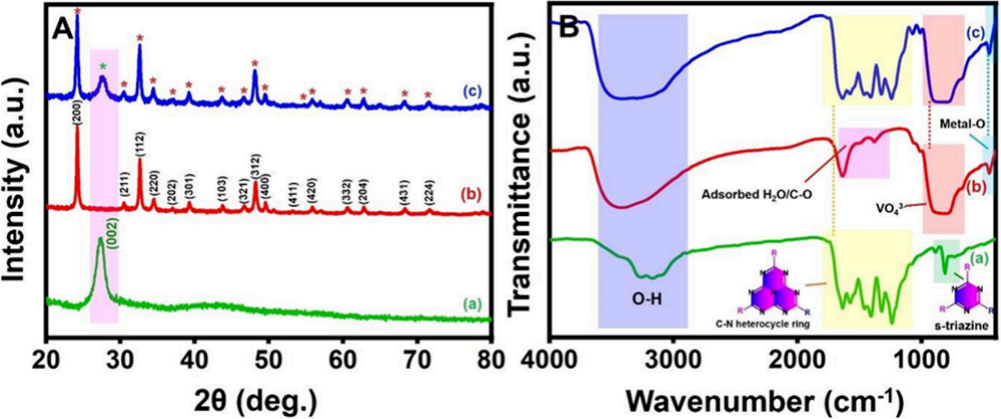
(A) XRD patterns and (B) FTIR spectra of the (a) P-CN, (b) HoVO_4_, and (c) HoVO_4_/P-CN nanocomposite.

The synthesized P-CN, HoVO_4_, and HoVO_4_/P-CN nanocomposites were examined through a series of characterization methods. The crystallographic structure of HoVO_4_, P-CN, and HoVO_4_/P-CN was analyzed by the X-ray diffraction (XRD) technique, as depicted in Figure 1A. The diffraction peak at a 2θ value of 24.7° in Figure 1A(a) is assigned to the (0 0 2) lattice plane of P-CN, which is in agreement with the literature.^49^ The formation of this structure can increase the interlayer spacing of P-doped CN, which could provide more active sites and surface irregularities to improve electrochemical activity.^50^ In addition, it increases the density of the states closer to the Fermi level, improves conductivity, and facilitates the charge transfer at the electrode/electrolyte interface. In Figure 1A(b), the diffraction peaks at 24.2, 30.4, 32.6, 34.4, 37.0, 39.2, 43.7, 46.6, 48.1, 49.5, 53.2, 55.8, 60.5, 62.7, 68.2, and 71.6° are assigned to the (2 0 0), (2 1 1), (1 1 2), (2 2 0), (2 0 2), (3 0 1), (1 0 3), (3 2 1), (3 1 2), (4 0 0), (4 1 1), (4 2 0), (3 3 2), (2 0 4), (4 3 1), and (2 2 4) lattice planes of tetragonal HoVO_4_, respectively, according to the JCPDS card no. 34-0421 of HoVO_4_.^39^ The XRD pattern of the HoVO_4_/P-CN nanocomposite in Figure 1A(c) displays every diffraction peak of HoVO_4_ and P-CN, confirming the successful loading of HoVO_4_ on the surface of P-CN without hampering its original structure. The absence of any impurity peaks in Figure 1A(c) demonstrates the formation of a high-purity HoVO_4_/P-CN nanocomposite. Such results show the efficacious formation of the HoVO_4_/P-CN nanocomposite.

Fourier transform infrared (FTIR) spectroscopy is employed to analyze the surface functional groups of the as-prepared samples, and related outcomes are depicted in Figure 1B. In Figure 1B(a), the characteristic absorption peaks at 1200–1600 cm^–1^ are attributed to the stretching vibration modes of CN heterocycles in the P-CN. The sharp absorption peak at 810 cm^–1^ is accredited to the out-of-plane breathing vibration of tri-*s*-triazine units in CN heterocycles.^47^ The wide absorption peak at 3000–3500 cm^–1^ is ascribed to its residual N–H component and O–H band, which are allied with uncondensed amino groups and surface-adsorbed water molecules.^49^ Notably, no vibration or stretching modes related to the P element are observed owing to the overlapping of the C–N bond vibration. For HoVO_4_, strong peaks at 830 and 455 cm^–1^ in Figure 1B(b) are attributed to the V–O (from the VO_4_^3–^ group) and the M–O stretching vibration. The weak peaks located at 3423 and 1637 cm^–1^ are ascribed to the bending vibration of water molecules. In Figure 1B(c), all the main characteristic peaks of HoVO_4_ and P-CN are well present in the FTIR spectrum of the HoVO_4_/P-CN nanocomposite, obviously confirming the successful formation of the HoVO_4_/P-CN nanocomposite, which is in agreement with the XRD characterization results.

**Figure 2:**
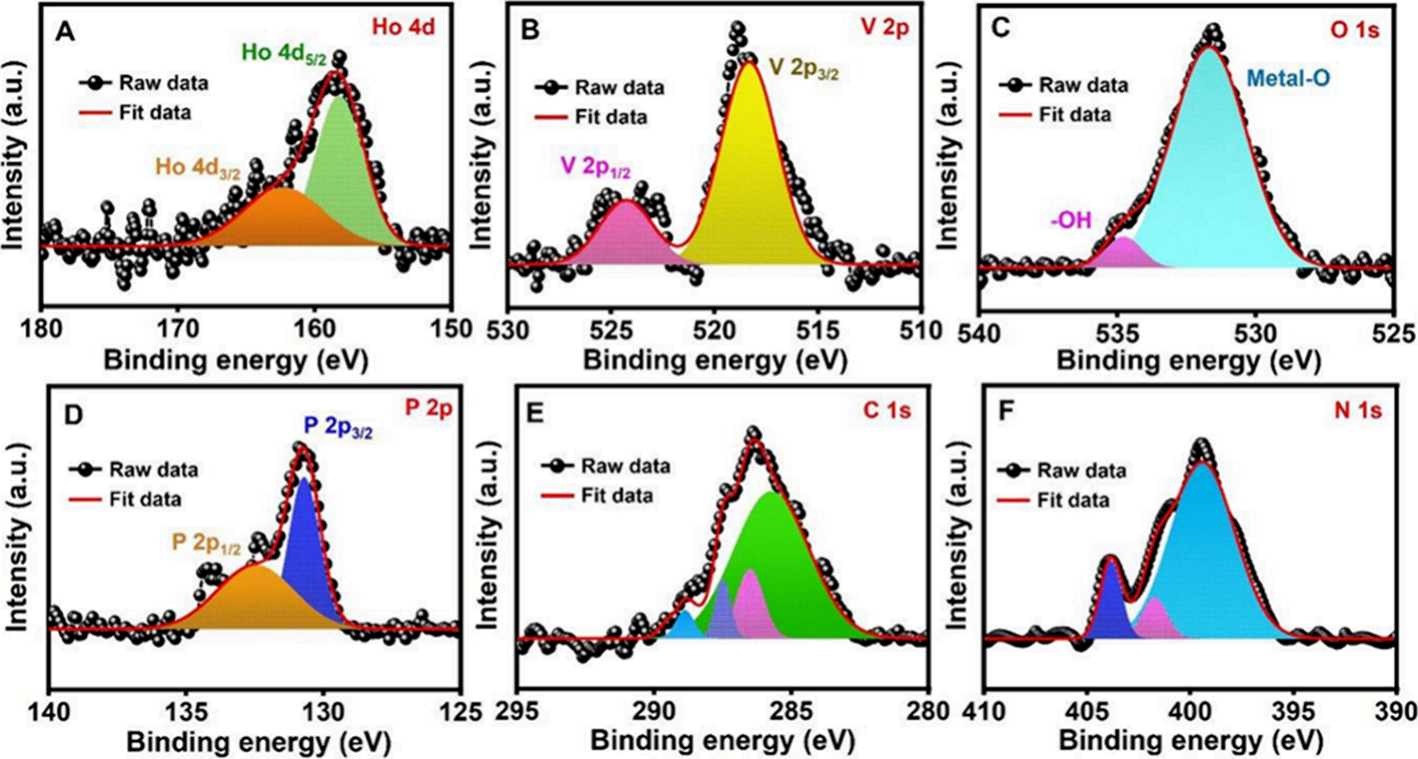
High-resolution XPS spectra of the HoVO_4_/P-CN nanocomposite for (A) Ho 4d, (B) V 2p, (C) O 1s, (D) P 2p, (E) C 1s, and (F) N 1s elements.

X-ray photoelectron spectroscopy (XPS) is used to analyze the surface chemical compositions and the valence states of the HoVO_4_/P-CN nanocomposite. The XPS survey spectrum in Figure S1 reveals the presence of Ho 4d, V 2p, O 1s, C 1s, N 1s, and P 2p elements on the surface of the HoVO_4_/P-CN nanocomposite. The high-resolution XPS spectrum of Ho 4d in Figure 2A demonstrates two peaks at 162.4 and 158.5 eV, which are assigned to Ho 4d_3/2_ and Ho 4d_5/2_, respectively.^39^ In Figure 2B, the peaks of V 2p at binding energies of 524.28 and 518.3 eV are indexed to V 2p_1/2_ and V 2p_3/2_ oxidation states, respectively. Figure 2C shows the O 1s XPS spectrum with peaks at 534.8 and 531.6 eV, which are attributable to vacancy oxygen (V–O) and lattice oxygen, respectively. The high-resolution spectrum of P 2p in Figure 2D can be curve-fitted with peaks at 132.54 and 130.72 eV corresponding to P–O and P–N/P–O–H, respectively. This result indicates the replacement of carbon atoms by phosphorus atoms and the formation of covalent bonds with nitrogen atoms adjacent to the heptazine unit.^50^ The high-resolution C 1s XPS spectrum in Figure 2E is fitted into four peaks at 288.8, 287.5, 286.5, and 285.7 eV, corresponding to the chemical bonds of C=N, N_2_–C= N, C–N–C, and C–N, respectively. The high-resolution XPS spectrum of N 1s in Figure 2F displays peaks at 409.85, 401.74, and 399.43 eV, which correspond to N–(C_3_), −N–P–, and N–C, respectively. These XPS analysis results confirm that HoVO_4_ and P-CN are successfully combined by chemical interaction.

**Figure 3:**
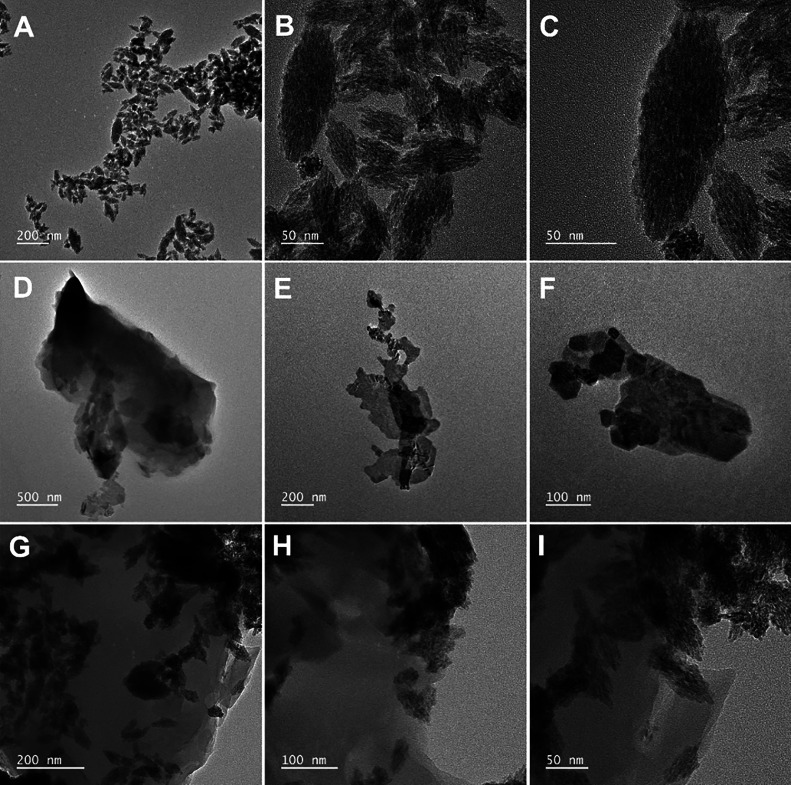
TEM images of the (A–C) HoVO_4_, (D–F) P-CN, and (G–I) HoVO_4_/P-CN nanocomposite.

Transmission electron microscopy (TEM) is used for observing the morphologies of HoVO_4_, P-CN, and HoVO4/P-CN. Figure 3A–C shows that the prepared HoVO_4_ has a nanorice morphology with abundant interior cavities and a length of 50 to hundreds of nanometers in range. The HoVO_4_ nanostructure possesses a surface with more active sites to promote electrochemical performance. In Figure 3D–F, the morphology of P-CN displays an ultrathin two-dimensional (2D) nanosheet-like structure. The P-CN has a 2D network structure that can enhance the mass transfer process and provide large-scale specific surface areas to ensure good dispersion of active sites. The TEM images of the HoVO_4_/P-CN nanocomposite in Figure 3G–I show that HoVO_4_ nanorices are covered on ultrathin P-CN nanosheets, and the loading of P-CN does not affect the distribution and orientation of HoVO_4_. The HAADF-STEM image in Figure S2A shows that the well-dispersed HoVO_4_ nanorices are tightly anchored onto the P-CN nanosheets. The energy-dispersive spectroscopy (EDS) spectrum in Figure S2B indicates the existence of holmium (Ho), vanadium (V), oxygen (O), phosphorus (P), carbon (C), and nitrogen (N) elements in the HoVO_4_/P-CN nanocomposite. The elemental mapping images in Figure S2C–H clearly demonstrate the corresponding distribution of Ho, V, and O elements in HoVO_4_ nanorices and the P, C, and N elements in P-CN nanosheets. The above physical characterization results confirm the successful preparation of the HoVO_4_/P-CN nanocomposite without any impurity.

### 3.2 Electrochemical Measurement

#### 3.2.1 Electrochemical Properties of Unmodified and Modified Electrodes

**Figure 4:**
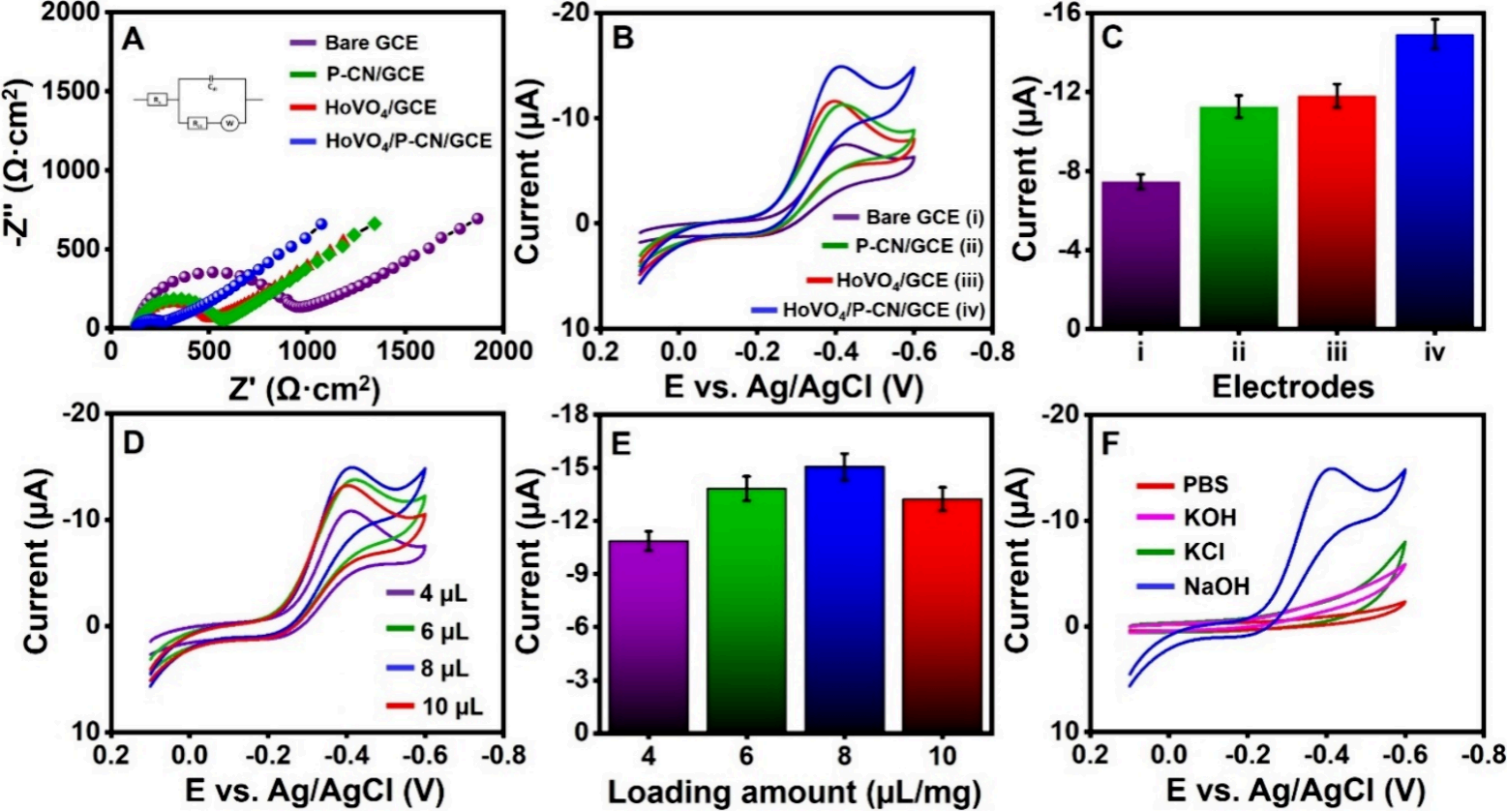
(A) Nyquist plots of different electrodes (inset: Randle’s equivalent circuit model). (B) CV curves of 90 μM H_2_O_2_ in 0.1 M NaOH on different electrodes at a scan rate of 50 mV/s. (C) Comparison of reduction current for different electrodes. (D) CV curves of 90 μM H_2_O_2_ on the HoVO_4_/P-CN/GCE prepared with different loading amounts of the HoVO_4_/P-CN nanocomposite. (E) Dependence of reduction current on the loading amount. (F) CV curves of 90 μM H_2_O_2_ in different electrolytes on the HoVO_4_/P-CN/GCE.

The interfacial charge transfer properties of various modified electrodes are evaluated through electrochemical impedance spectroscopy (EIS).^27^Figure 4A shows the Nyquist plots of the bare GCE, HoVO_4_/GCE, P-CN/GCE, and HoVO_4_/P-CN/GCE in a 0.1 M KCl solution, which contains 5 mM [Fe(CN)_6_]^3–/4–^. These Nyquist plots are fitted using Randle’s equivalent circuit model shown in the inset of Figure 4A. Randle’s equivalent circuit model consists of charge transfer resistance (*R*_ct_), solution resistance (*R*_s_), double-layer capacitance (*C*_dl_), and Warburg impedance (*Z*_w_). The *R*_ct_ value of the electrode surface is reflected in the semicircular diameter in the Nyquist plot. The equivalent circuit calculation shows that the *R*_ct_ values of bare GCE, P-CN/GCE, HoVO_4_/GCE, and HoVO_4_/P-CN/GCE are 831, 447, 330, and 157 Ω, respectively. The HoVO_4_/P-CN/GCE possesses the lowest *R*_ct_ value, which indicates the fastest electron transfer, the best conductivity, and excellent catalytic ability among all electrodes. Therefore, HoVO_4_/P-CN/GCE is desirable for further electrochemical investigation toward H_2_O_2_ detection.

#### 3.2.2 Electrochemical Sensing of H_2_O_2_

The cyclic voltammetry (CV) method is used to evaluate the electrochemical behavior of different electrodes. Figure 4B illustrates the CV comparison of nonenzymatic analysis of 90 μM H_2_O_2_ in 0.1 M NaOH on the bare GCE, HoVO_4_/GCE, P-CN/GCE, and HoVO_4_/P-CN/GCE at a scan rate of 50 mV/s. The CV curve of HoVO_4_/P-CN/GCE exhibits a distinct reduction response of H_2_O_2_ with the lowest peak potential of −0.412 V (vs Ag/AgCl) compared to other electrodes, such as the bare GCE, HoVO_4_/GCE, and P-CN/GCE. Notably, no oxidation peak occurs during the electrochemical reaction, suggesting an irreversible reduction process. Based on the above experimental results, a possible electrochemical reaction mechanism of H_2_O_2_ on HoVO_4_/P-CN/GCE is shown in Figure S3A. The high electrocatalytic activity of the HoVO_4_/P-CN nanocomposite might be mainly attributed to the high effective surface area, good chemical stability, high electronic conductivity, synergistic effect, unique morphology, enhanced electron transfer, and abundant electroactive sites. The reduction currents for the HoVO_4_/P-CN/GCE are 1.26-, 1.32-, and 2-fold greater than those for the P-CN/GCE, HoVO_4_/GCE, and bare GCE, respectively. Figure 4C displays the bar diagram of the reduction current of different electrodes. Thus, HoVO_4_/P-CN/GCE with the highest current response and the lowest peak potential is selected for further electrochemical analysis of H_2_O_2_.

#### 3.2.3 Influences of the Loading Amount and Supporting Electrolyte

The optimal loading amount of the HoVO_4_/P-CN nanocomposite on the GCE is essential for enhancing the electrochemical reaction for H_2_O_2_ detection. In this study, the HoVO_4_/P-CN/GCEs prepared with volumes of HoVO_4_/P-CN suspension of 4, 6, 8, and 10 μL were examined by the CV method in the presence of 90 μM H_2_O_2_, as shown in Figure 4D,E. When the HoVO_4_/P-CN’s loading amount increases from 4 to 8 μL, the current response initially increases and achieves the maximum value at 8 μL. The further increase in the loading amount to 10 μL results in the decreased peak reduction current due to high mass transfer resistance. Therefore, 8 μL of the HoVO_4_/P-CN suspension is the optimized loading amount on the surface of the GCE for electrochemical detection of H_2_O_2_. The impact of the supporting electrolytes on the electrochemical reaction of 90 μM H_2_O_2_ on HoVO_4_/P-CN/GCE is shown in Figure 4F. The studied 0.1 M supporting electrolytes include PB solution, potassium hydroxide (KOH) solution, potassium chloride (KCl) solution, and sodium hydroxide (NaOH) solution. The electrochemical results show that the best electrochemical response is obtained for the NaOH solution. Thus, the NaOH solution is chosen as the supporting electrolyte in the subsequent electrochemical experiments for H_2_O_2_ detection.

#### 3.2.4 Influence of the Concentration and Scan Rate

The CV response of various concentrations of H_2_O_2_ on the HoVO_4_/P-CN/GCE is shown in Figure S3B. The peak reduction current increases significantly as the H_2_O_2_ concentration grows from 10 to 90 μM. A good linear relationship between the peak reduction current and the H_2_O_2_ concentration is found in Figure S3C. The linear regression equation is described as *I*_pc_ (μA) = −0.0809 [H_2_O_2_] (μM) – 7.6836 with a correlation coefficient of 0.994. These results demonstrate excellent electrocatalytic detection ability toward H_2_O_2_ by using the HoVO_4_/P-CN-modified electrode. The electrochemical reduction response of 90 μM H_2_O_2_ on the HoVO_4_/P-CN/GCE at different scan rates is explored by the CV method, as shown in Figure S3D. The peak reduction current increases gradually with the scan rate in the range from 0.02 to 0.2 V/s. Figure S3E shows that the peak reduction current linearly increases with the square root of the scan rate. The linear regression equation is described as *I*_pc_ (μA) = −21.036 [scan rate (V/s)]^1/2^ – 9.0267 with a correlation coefficient of 0.992. These results demonstrate that the electrocatalytic reduction behavior of H_2_O_2_ on the surface of HoVO_4_/P-CN/GCE is a diffusion-controlled process.

#### 3.2.5 Analytical Performance

**Figure 5:**
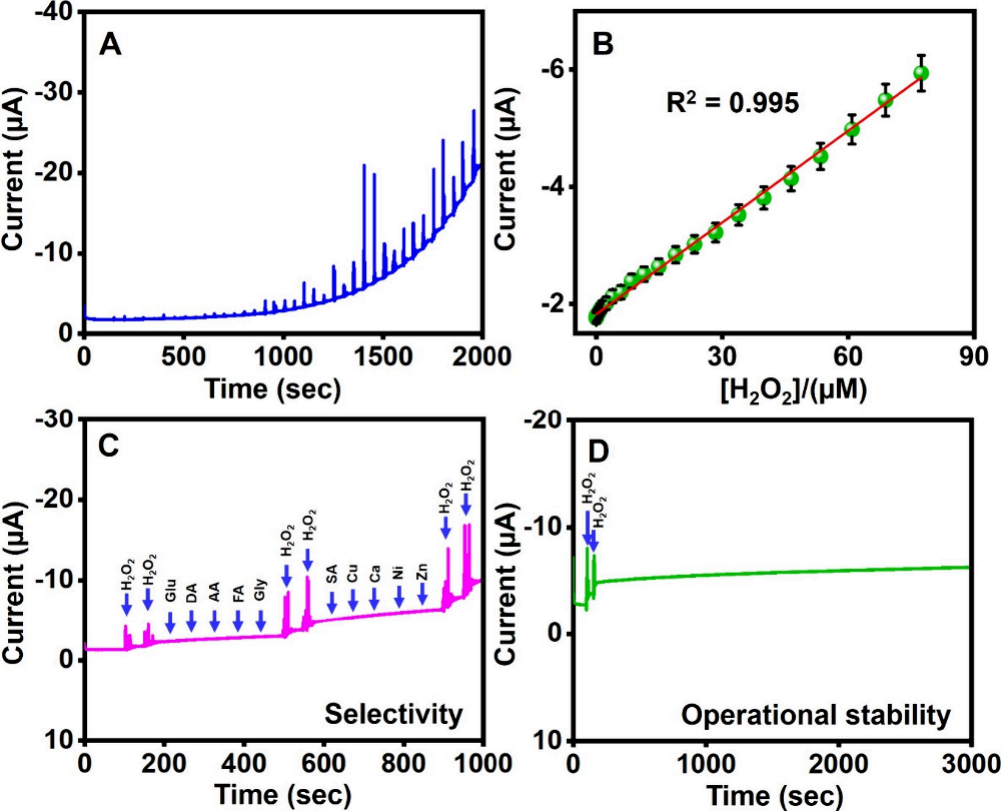
(A) Amperometric response of HoVO_4_/P-CN/GCE for the successive addition of different concentrations of H_2_O_2_ in 0.1 M NaOH. (B) Dependence of the peak current on the H_2_O_2_ concentration. (C) Amperometric response of HoVO_4_/P-CN/GCE for the addition of H_2_O_2_ with the presence of various interfering compounds. (D) Operational stability test of HoVO_4_/P-CN/GCE for the detection of 30 μM H_2_O_2_.

The amperometric method is used to investigate the analytical performance of HoVO_4_/P-CN/GCE for H_2_O_2_ detection by the consecutive addition of various concentrations of H_2_O_2_ under continuous stirring. It is noted from Figure 5A that the reduction current rises with the H_2_O_2_ concentration in the stair-step way and achieves the steady-state current within 3 s. Figure 5B shows that the reduction current has a good linear relationship with the H_2_O_2_ concentration in the range from 0.009 to 77.4 μM. The fitting linear regression equation is *I*_pc_ (μA) = −0.0504 [H_2_O_2_](μM) – 1.8432 with a correlation coefficient of 0.995. The LOD and the sensitivity of HoVO_4_/P-CN/GCE are calculated as 0.003 μM and 0.72 μA μM^–1^ cm^–2^, respectively. The analytical performance of the proposed HoVO_4_/P-CN-modified GCE for H_2_O_2_ detection is compared to the previous reports in Table 1.^11,51−60^ This analytical study reveals that the proposed HoVO_4_/P-CN nanocomposite-based electrochemical sensor shows superior or equivalent sensing performance for H_2_O_2_ detection compared with the formerly reported electrochemical platforms. The superior electrochemical performance of the HoVO_4_/P-CN nanocomposite is attributed to good electrical conductivity, synergistic effect, large surface area, and more active sites. Therefore, the HoVO_4_/P-CN nanocomposite-modified GCE can be utilized in an actual sample analysis.

**Table 1:** Performance Comparison of Various Electrode Materials for H_2_O_2_ Detection.

**electrode material**	**linear range (μM)**	**LOD (μM)**	**ref.**
CuO-CeO_2_/MXene	5–100	1.7	(11)
ERGO	1–16	0.7	(51)
Ag–Au NPs	30–300	0.18	(52)
Pd NP	0.1–1.8	0.04	(53)
hemin/PA-PANI	2–102	1.2	(54)
Fe_3_O_4_@Ag	0.5–20	0.16	(55)
Ag–TiN	0.2–220	0.02	(56)
CuO/g-C_3_N_4_	0.5–50	0.31	(57)
Pt_rGO@PILs	2.3–250	1.5	(58)
Nb_2_CT*_x_*/PB	1–10	0.2	(59)
	10–100		
Fe_3_O_4_/Gr	10–110	4.79	(60)
HoVO_4_/P-CN	0.009–77.4	0.003	this work

#### 3.2.6 Selectivity, Stability, Repeatability, and Reproducibility

Selectivity is essential in evaluating electrochemical sensors for H_2_O_2_ detection in practical applications. For this reason, the effect of other potentially similar or interfering compounds that might coexist in the actual samples containing H_2_O_2_ was examined. The selectivity of the HoVO_4_/P-CN-modified GCE is investigated by the amperometric method for the detection of 30 μM H_2_O_2_ with the continuous addition of various interfering compounds. The studied interfering compounds include the 100-fold excess amount of glucose (Glu), dopamine (DA), ascorbic acid (AA), folic acid (FA), glycine (Gly), and salicylic acid (SA) and the 200-fold excess amount of copper (Cu), calcium (Ca), nickel (Ni), and zinc (Zn). The current response shown in Figure 5C shows that the existence of potentially interfering compounds does not noticeably affect the reduction current produced by the HoVO_4_/P-CN-based electrochemical sensor. The above results indicate that the proposed HoVO_4_/P-CN/GCE exhibits a high selectivity for determining H_2_O_2_.

The operational stability of HoVO_4_/P-CN/GCE is investigated by the amperometry method. The investigation of Figure 5D shows that the current response for 30 μM H_2_O_2_ can remain at 94% of its original response after 3000 s, which reveals the preferable operational stability of the proposed HoVO_4_/P-CN sensor. Figure S4A shows the repeatability test of HoVO_4_/P-CN/GCE for the detection of 90 μM H_2_O_2_ under the same condition mentioned in Section 3.2.2. The histogram of peak current for four HoVO_4_/P-CN-based H_2_O_2_ sensors is shown in Figure S4B. The relative standard deviation (RSD) value of the peak current is 0.313%, indicating that the HoVO_4_/P-CN-modified GCE has good repeatability for H_2_O_2_ detection. The reproducibility of the proposed sensor is evaluated by detecting 90 μM H_2_O_2_ on three HoVO_4_/P-CN-based sensors under the same condition, as depicted in Figure S4C. The peak current histogram of the three HoVO_4_/P-CN-based sensors is shown in Figure S4D. The CV analysis results show an RSD value of 0.8%, which confirms that the HoVO_4_/P-CN-based sensor has good reproducibility for H_2_O_2_ detection. These results indicate satisfactory selectivity, stability, repeatability, and reproducibility of the HoVO_4_/P-CN-based electrochemical sensor for H_2_O_2_ sensing.

#### 3.2.7 Practical Application

The practicability of HoVO_4_/P-CN/GCE was investigated by the determination of H_2_O_2_ in real samples. The sample for milk was purchased from a local shop in Taipei, Taiwan. The sample for human urine was collected from a healthy volunteering person. These H_2_O_2_ free real samples were centrifuged and diluted with DI water. Then, both samples were spiked with the H_2_O_2_ solution according to the standard addition method. The amperometry response of HoVO_4_/P-CN/GCE in Figure S5A,B displays a robust and good current change for the detection of H_2_O_2_ in milk and human urine. The reliability of the proposed H_2_O_2_ sensor based on the HoVO_4_/P-CN nanocomposite was evaluated using the high-performance liquid chromatography (HPLC) method. The recovery values measured by the proposed electrochemical method and the HPLC method are given in Table S1 for comparison. The recovery values for milk and human urine samples measured by the proposed electrochemical sensor are in the range of 96.7–98.75%, which is close to the range (97.3–98.6%) measured by the HPLC analysis. This indicates that the proposed HoVO_4_/P-CN nanocomposite-based electrochemical sensor has good accuracy and satisfactory reliability for the detection of H_2_O_2_ in the actual samples.

## 4 Conclusions

We present the synthesis of HoVO_4_/P-CN nanocomposites as an efficient electrocatalyst for nonenzymatic electrochemical H_2_O_2_ detection by attaching the hydrothermally synthesized HoVO_4_ nanorices on ultrathin P-CN nanosheets. Various analytical methods, such as XRD, FTIR, XPS, TEM, EDS, and elemental mapping, are used to confirm the successful formation of the HoVO_4_/P-CN nanocomposites. The HoVO_4_/P-CN-modified GCE exhibits superior electrochemical sensing capability toward H_2_O_2_ reduction compared to the bare GCE, HoVO_4_-modified GCE, and P-CN-modified GCE. The excellent detection performance of the proposed H_2_O_2_ sensor includes the wide linear working range of 0.009–77.4 μM, ultralow LOD of 3 nM, and high sensitivity of 0.72 μA μM^–1^ cm^–2^. In addition, the HoVO_4_/P-CN-modified GCE exhibits satisfactory stability, repeatability, and reproducibility. Importantly, the HoVO_4_/P-CN-modified GCE has high selectivity in the presence of various potential interfering compounds in real-world samples, such as milk and human urine. The proposed electrochemical sensor based on HoVO_4_/P-CN nanocomposites presents great promise for nonenzymatic sensing of H_2_O_2_ in the fields of food safety and clinical diagnostics.

## Supporting Information Available

Materials, instrumentation, synthesis of HoVO_4_ nanorices, synthesis of P-CN nanosheets, XPS survey spectrum, HAADF-STEM image, EDS spectrum, elemental mapping images, electrochemical reduction mechanism of H_2_O_2_, CV curves of different concentrations of H_2_O_2_ on the HoVO_4_/P-CN/GCE, dependence of peak current on the H_2_O_2_ concentration, CV response of 90 μM H_2_O_2_ on the HoVO_4_/P-CN/GCE at different scan rates, dependence of peak current on the square root of scan rates, repeatability test, reproducibility test, amperometric response for the addition of H_2_O_2_ in milk and human urine samples, and recovery measurement result (PDF)
