# Supplementary material for: Synergism of Holmium Orthovanadate/Phosphorus-Doped Carbon Nitride Nanocomposite: Nonenzymatic Electrochemical Detection of Hydrogen Peroxide
Source: Inorg Chem. 2024 Jan 29;63(6):3019–27. doi: 10.1021/acs.inorgchem.3c03804 (PMC10865356; doi:10.1021/acs.inorgchem.3c03804)
Supplement: Supplementary file 1 — ic3c03804_si_001.pdf [file ic3c03804_si_001.pdf]

# ***Supporting Information***

## Synergism of Holmium Orthovanadate/Phosphorus-Doped Carbon Nitride Nanocomposite: Non-Enzymatic Electrochemical Detection of Hydrogen Peroxide

***Thangavelu Kokulnathan[a], Tzyy-Jiann Wang[a*], Faheem Ahmed[b], Thamraa Alshahrani[c], Nishat Arshi[d]***

[a]*Department of Electro-Optical Engineering, National Taipei University of Technology, Taipei 106, Taiwan.*

[b]*Department of Applied Sciences & Humanities, Faculty of Engineering & Technology, Jamia Millia Islamia, New Delhi-110 025, India.*

[c]*Department of Physics, College of Science, Princess Nourah bint Abdulrahman University, P.O. Box 84428, Riyadh 11671, Saudi Arabia.*

[d]*Department of Basic Sciences, Preparatory Year Deanship, King Faisal University, P.O. Box-400, Al-Ahsa 31982, Saudi Arabia.*

*** Corresponding Author**

E-mail: f10939@ntut.edu.tw

***Materials***

Holmium (III) nitrate pentahydrate ($Ho(NO_3)_3 \cdot 5H_2O$; 99.99%), ammonium metavanadate ($NH_4VO_3$; ≥99%), sodium hydroxide (NaOH; ≥98% (anhydrous)), ammonium dihydrogen phosphate ($NH_4H_2PO_4$; ≥98%), hydrogen peroxide ($H_2O_2$; 35%), melamine ($C_3H_6N_6$; 99%), and other chemicals were purchased from Sigma-Aldrich (Taiwan). All the materials were used as received without further purification. All aqueous solutions were prepared using deionized (DI) water.

***Instrumentation***

X-ray diffraction (XRD) analysis was carried out using a Bruker AXS D8 advance X-ray diffractometer with the Cu-Kα radiation of wavelength 1.540 Å. Fourier-transform infrared (FT-IR) spectroscopy was performed using a PerkinElmer Frontier FT-IR spectrometer. The X-ray photoelectron (XPS) spectra were acquired using a JEOL JPS-9030 spectrometer. Transmission electron microscopy (TEM) images were recorded by a JEOL JEM-2100F system equipped with a high-angle annular dark-field scanning transmission electron microscopy (HAADF-STEM), elemental mapping function, and energy-dispersive X-ray spectroscopy (EDS). Electrochemical impedance spectroscopy (EIS) was conducted on a Metrohm Autolab NOVA 2 system. A signal amplitude of 5 mV was applied in the frequency ranging from 0.01 Hz to 100 kHz. High performance liquid chromatography (HPLC) was analyzed by Vanquish Flex UHPLC. A conventional three-electrode electrochemical system used for the electrochemical measurement consists of the CHI electrochemical workstation, a glassy carbon electrode (GCE; 0.07 $cm^2$) participating as the working electrode, a platinum wire acting as the counter electrode, and Ag/AgCl (Sat. KCl) acting as the reference electrode.

***Synthesis of $HoVO_4$ nanorices***

The nanorice like $HoVO_4$ was synthesized by the hydrothermal method. In detail, 0.468 g of $NH_4VO_3$ was dissolved in 40 mL of DI water at 60 °C under constant magnetic stirring. After 15 min, 0.96 g of NaOH was added into the above solution at 60 °C under constant magnetic stirring to obtain solution A. 1.76 g of $Ho(NO_3)_3 \cdot 5H_2O$ was dissolved in 40 mL of DI water under constant magnetic stirring for 30 min to obtain solution B. Next, solution B was slowly poured into solution A under constant stirring for 30 min to yield a mixed homogeneous solution. This mixed homogeneous solution was transferred into a 100 mL

Teflon-lined stainless-steel autoclave and then heat-treated at 180 °C for 12 hr. After cooling, the obtained precipitate was centrifuged, washed with ethanol/DI water, and dried in an oven at 60 °C. The resultant yellowish product was calcined at 600 °C for 3 hr with a heating rate of 5 °C $min^{-1}$ to form nanorice like $HoVO_4$.

***Synthesis of P-CN nanosheets***

The P-CN nanosheets were prepared by the following process. A mixture of $NH_4H_2PO_4$ and $C_3H_6N_6$ was dispersed in 50 mL of DI water at room temperature by ultrasonication for 90 min to obtain a homogeneous suspension. The resultant precursor material was thoroughly washed with DI water and dried in an oven at 50 °C overnight. The prepared material was well-grounded in a mortar and then calcined in the muffle furnace at 550 °C for 3 hr. Finally, the obtained precipitate was centrifuged, washed, and dried in an oven at 60 °C.

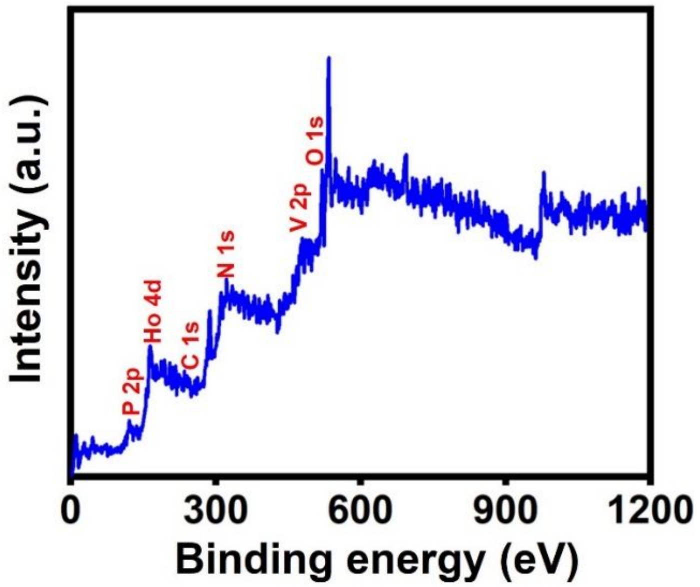


**Fig. S1** XPS survey spectrum of $HoVO_4$/P-CN nanocomposite.

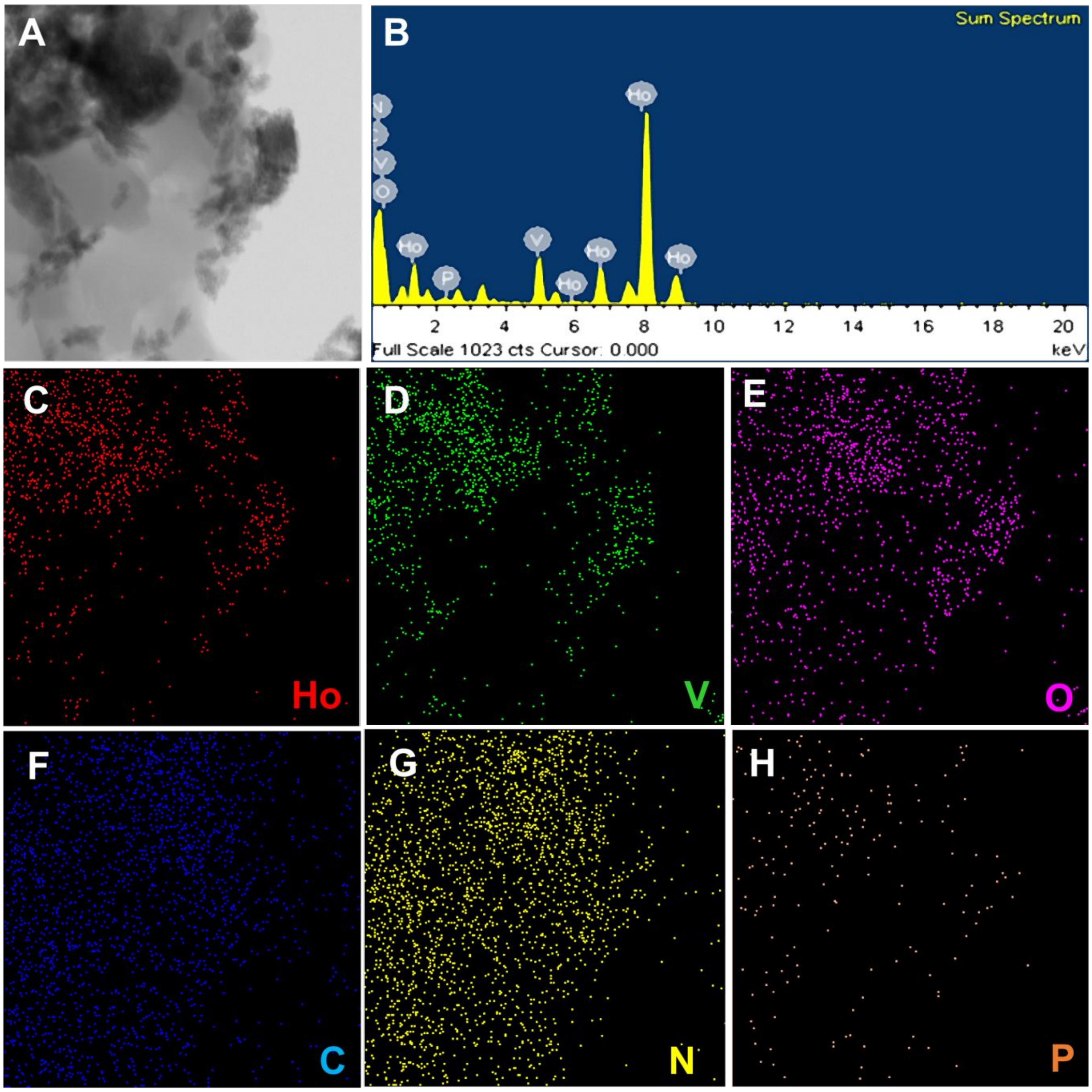


**Fig. S2** (A) HAADF-STEM image and (B) EDS spectrum of $HoVO_4$/P-CN nanocomposite. (C–H) Corresponding elemental mapping images for the Ho, V, O, C, N, P elements.

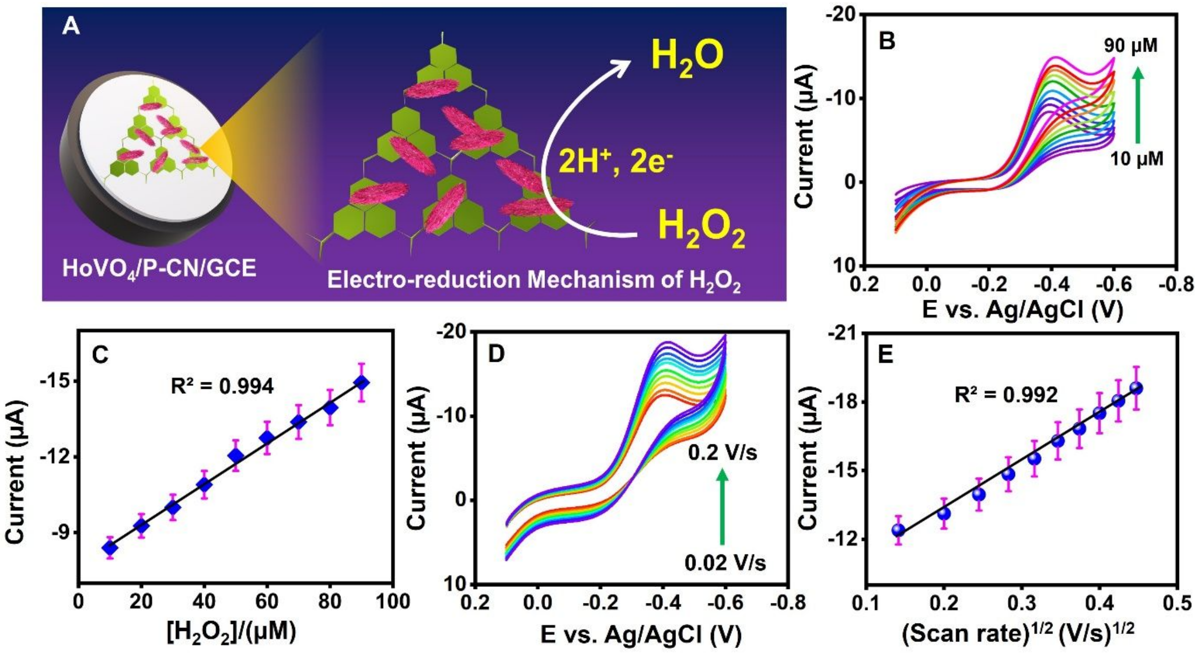


**Fig. S3** (A) Electrochemical reduction mechanism of $H_2O_2$ on the $HoVO_4$/P-CN/GCE. (B) CV response of different concentrations of $H_2O_2$ on the $HoVO_4$/P-CN/GCE. (C) Dependence of peak current on the $H_2O_2$ concentration. (D) CV response of 90 μM $H_2O_2$ on the $HoVO_4$/P-CN/GCE at different scan rates. (E) Dependence of peak current on the square root of scan rate.

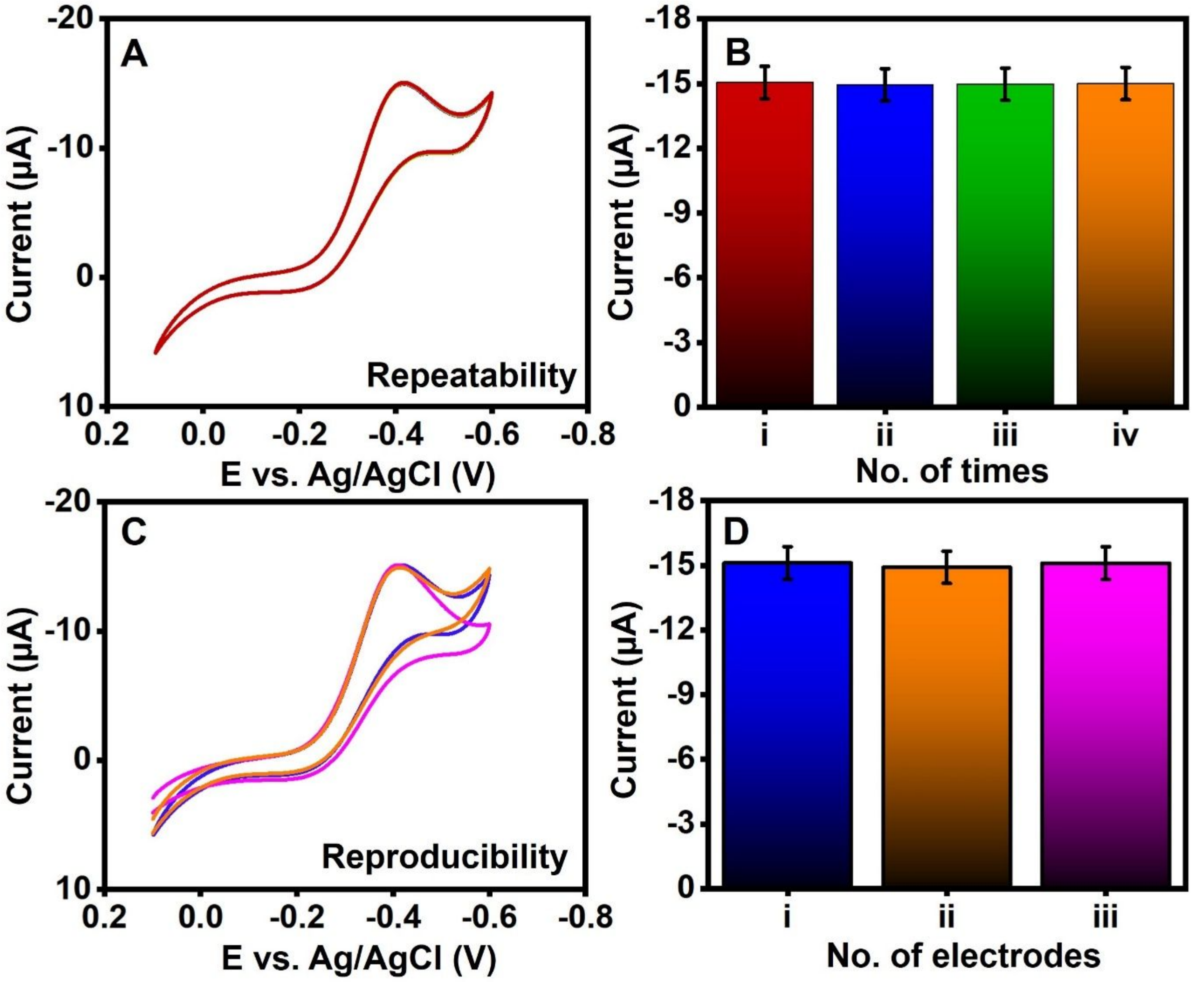


**Fig. S4** (A, B) Repeatability test and (C, D) reproducibility test of $HoVO_4$/P-CN/GCE for the detection of 90 μM $H_2O_2$.

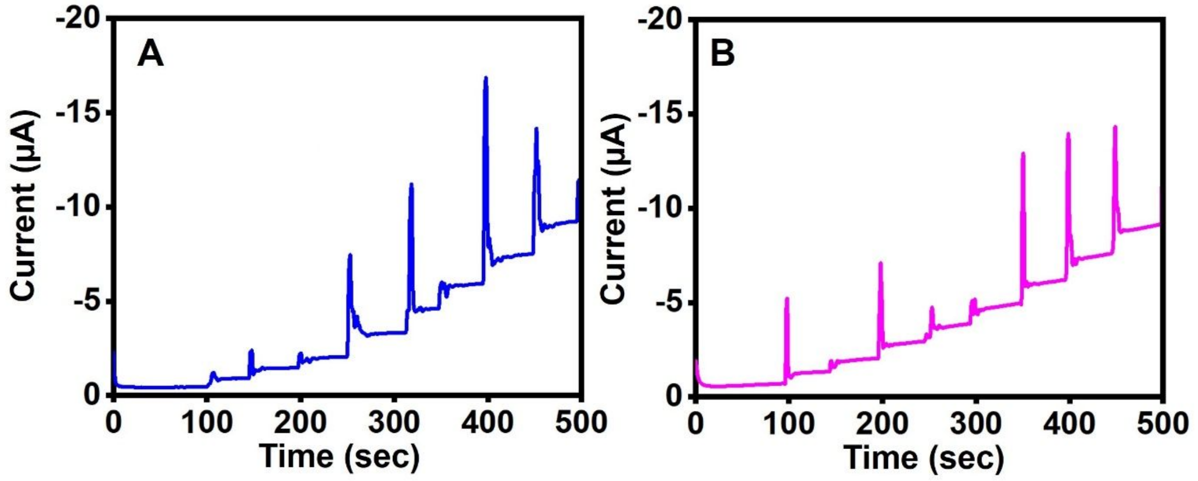


**Fig. S5** Amperometric response for the addition of $H_2O_2$ (0.16–7.0 µM) in (A) milk and (B) human urine samples on the $HoVO_4$/P-CN/GCE.

**Table S1**. Recovery measurement result of $H_2O_2$ in milk and human urine samples.

| Sample | Added (µM) | Electrochemical sensor | | HPLC analysis | |
|---|---|---|---|---|---|
| | | Found (µM) | Recovery (%) | Found (µM) | Recovery (%) |
| Milk | 0 | Not found | – | Not found | – |
| | 0.16 | 0.158 | 98.75 | 0.156 | 97.50 |
| | 0.30 | 0.290 | 96.70 | 0.292 | 97.30 |
| | 0.50 | 0.490 | 98.00 | 0.493 | 98.60 |
| Human urine | 0 | Not found | – | Not found | – |
| | 0.16 | 0.156 | 97.50 | 0.157 | 98.10 |
| | 0.30 | 0.295 | 98.30 | 0.294 | 98.00 |
| | 0.50 | 0.485 | 97.00 | 0.488 | 97.60 |
